# From our readers

**Published:** 2012

**Thank you to all our readers who wrote in to share stories and experiences around low vision**.

**QUESTIONS FROM MADAGASCAR**

We received several questions by **Lala Jaona landrinirina** and **Verohanitra Raharimamonjy** of the Centre National de Formation en Santé Oculaire Communautaire in Madagascar. The answers are by **Clare Gilbert, Karin van Dijk**, and **Sumrana Yasmin**.

**How can we persuade ophthalmologists and parents that it is important to refer children for low vision services?**

- During the eye examination, show parents what their child can currently see, and what they are likely to be able to see after refraction and low vision support. For example, reading a poster on the wall or text in a school book
- Include simulation of low vision (for example, looking through a plastic sheet) when training eye care practitioners
- Find children with low vision who are willing to explain the difference that low vision services have made to them. They can speak with parents, ophthalmologists and others who need convincing
- People working in low vision can talk to their colleagues in clinical eye care about the importance of low vision care. Use real examples of children who benefited.

**How can we persuade people with low vision to accept their condition?**

- People who have lost much of their sight will go through a process of grieving, consisting of several stages, including denial, anger, bargaining, and depression; acceptance is the final stage. This process takes time and cannot be hurried. Be there, listen, and provide what support you can
- Put the person in contact with someone of similar age who has accepted the situation and has benefited from low vision services.
  **How can we convince children to use a low vision device?**
- Start with tasks that are fun to do. Play a game with the child, using the device
- Avoid using just an acuity chart! Use real tasks the child is interested in, such as looking at a flower or a family photo, and which they would now struggle to do without magnification
- Show the child that they can do the same activities as their peers, such as reading on their own, if they use a device
- Provide good training, both for the child and the family: first in private so the child can practise without fear of teasing; later, in a variety of settings
- Organise a meeting with a child who successfully uses a magnifying device
- Tell the teacher why a child needs to use a certain device and for what tasks
- Help the teacher to create a positive atmosphere in the classroom. It is helpful if classmates can try out the device and see for themselves why it is useful.

**What devices are best suited to children?**

Here are some tips. Each child is different!

- Where possible, first make tasks bigger, brighter, and bolder before introducing magnifiers
  •Younger children might find it easier to learn to use a dome or stand magnifier first as they do not have to hold the magnifier off the page. Try high-plus spectacles and/or hand magnifiers later
- Older children do not want to be different from their peers, so pocket magnifiers might be liked as one can quickly put them in one's pocket when not in use.
  **How can we keep a child's attention during an examination?**
- For practical tips, see ‘When your eye patient is a child’ (Community Eye Health J 2010; 23(72): 4–11). Available on CD or www.cehjournal.org/journal.html

**LOW-COST PEN-MOUNTED CCTV CAMERA**

Using locally available CCTV cameras, used for surveillance in banks or offices, **V Srinivasan** and his team at Aravind Eye Hospital have built a magnification system that can be connected to a normal television. The system costs approximately US $60 to make and can be adapted to connect to a laptop computer. It is also possible to use a very small CCTV camera mounted on a pen, allowing the user to see the writing magnified on screen. For copies of plans and more information on how to produce such a system, write to Prof V Srinivasan, LAICO, 72, Kuruvikaran Salai, Gandhi Nagar, Madurai 625 020, Tamil Nadu, India. Email: v.srinivasan@aravind.org

**Figure:**
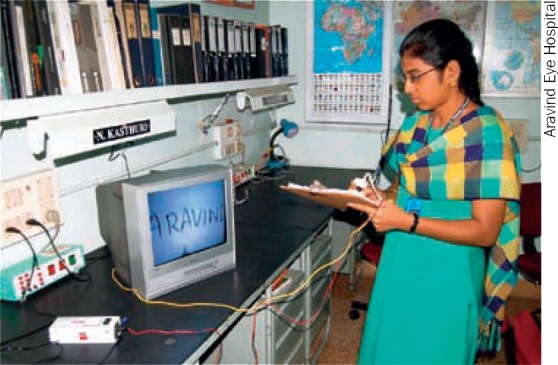

**VIEW FROM NIGERIA**

**Enechi Gilbert**, National Secretary of the Low Vision and Rehabilitation Society of Nigeria (LVRSN), wrote to say that there is still a big gap between the need for low vision services and the country's ability to provide them, in large part because of a lack of low vision professionals. He feels all eye care workers should take responsibility for low vision: “Every eye care professional should be able to at least detect and then diagnose, manage, or refer (in accordance to his/her professional abilities and limitations) people with low vision to other established low vision practitioners or low vision centers.”

**REFRACTIVE ERROR AND LOW VISION**

Two readers reported that many children were referred to low vision services before they had received adequate refractive error services.

**Vicky Hopley, in Madagascar**, looked at the records kept by Madagascar's first low vision clinic during its first year of operation. She found that, of the 65 children seen in 2008, 15 (around 23%) had uncorrected refractive error.

**K Sapkota, in Nepal**, examined the patient records of the low vision clinic in Nepal Eye Hospital over a 16-month period, ending October 2011. Of the 69 children examined, general spectacles improved the visual acuity of 52 children (75%).

**Our consulting editors respond:** Even if someone has low vision, their refractive error must still be corrected. The right pair of spectacles will improve the vision of people with low vision, even if it doesn't bring their vision back to ‘normal’. Many people with low vision will not have received good refractive correction before they are seen in the low vision clinic. These findings emphasise the importance of refraction during any low vision assessment.

